# Cerebrovascular stenosis and mortality after isolated off-pump coronary artery bypass grafting

**DOI:** 10.1186/s13019-026-04664-y

**Published:** 2026-07-29

**Authors:** YuKang Pei, Chen Bai, JianGang Wang

**Affiliations:** 1 Department of Valve and Atrial Fibrillation Surgery, Beijing Anzhen Hospital, Capital Medical University, Beijing, China; 2 Department of Cardiovascular Surgery, Beijing Anzhen Hospital, Capital Medical University, No. 2 Anzhen Road, Chaoyang District, Beijing, 100029 China

**Keywords:** Cerebrovascular stenosis, Vertebral artery stenosis, Carotid artery stenosis, Off-pump coronary artery bypass grafting, Mortality.

## Abstract

**Background:**

Cerebrovascular atherosclerosis is common in patients undergoing coronary revascularization. Although carotid artery disease has been extensively studied, the association between vertebral artery stenosis and long-term outcomes after isolated off-pump coronary artery bypass grafting (OPCABG) remains uncertain.

**Methods:**

We performed a retrospective cohort study of consecutive patients undergoing isolated OPCABG at a tertiary cardiac center between January 2018 and July 2021. Preoperative bilateral carotid and vertebral artery duplex ultrasonography was reviewed. Severe cerebrovascular stenosis was defined as 70%–99% luminal narrowing or complete occlusion involving at least one extracranial carotid or vertebral artery. The primary outcome was long-term all-cause mortality. Kaplan–Meier analysis and multivariable Cox proportional hazards regression were used to evaluate associations with mortality. In-hospital postoperative cerebral infarction and 30-day all-cause mortality were additionally summarized.

**Results:**

Among 1,277 patients, 186 (14.6%) had severe cerebrovascular stenosis, including 100 with severe carotid disease and 109 with severe vertebral disease; 23 patients had both. During a median follow-up of 4.79 years, 87 deaths occurred. Severe carotid stenosis was associated with worse unadjusted survival (log-rank *P* = 0.028) but was not independently associated with mortality after multivariable adjustment (hazard ratio [HR], 1.35; 95% confidence interval [CI], 0.86–2.13; *P* = 0.196). Severe vertebral stenosis (HR, 1.86; 95% CI, 1.22–2.84; *P* = 0.004) and any severe cerebrovascular stenosis (HR, 1.69; 95% CI, 1.21–2.37; *P* = 0.002) were independently associated with mortality. In-hospital postoperative cerebral infarction (5.91% vs. 0.55%; *P* < 0.001) and 30-day mortality (4.84% vs. 1.65%; *P* = 0.011) were more frequent among patients with severe cerebrovascular stenosis.

**Conclusions:**

Severe cerebrovascular stenosis was associated with increased long-term mortality after isolated OPCABG. Severe vertebral artery stenosis showed the strongest association and may represent a marker of more extensive systemic atherosclerotic disease. These findings support consideration of both carotid and vertebral territories during preoperative vascular assessment, while prospective studies are needed to determine clinical utility.

**Supplementary Information:**

The online version contains supplementary material available at https://doi.org/10.1186/s13019-026-04664-y.

## Introduction

Off-pump coronary artery bypass grafting (OPCABG) is an established revascularization strategy for patients with advanced coronary artery disease. Despite improvements in surgical technique and perioperative care, long-term outcomes after coronary revascularization remain influenced by age, ventricular function, metabolic disease, renal dysfunction, and the overall burden of systemic atherosclerosis.

Cerebrovascular atherosclerosis frequently coexists with coronary artery disease, and preoperative vascular assessment in cardiac surgery has traditionally focused on the carotid arteries [1–4]. Carotid stenosis has been studied primarily in relation to perioperative stroke and short-term outcomes after coronary artery bypass grafting (CABG) [5–9]. However, carotid disease represents only one component of the extracranial cerebral circulation.

Vertebral artery disease has received substantially less attention in the cardiac surgical literature [2, 3, 10]. Because vertebral atherosclerosis may reflect diffuse vascular disease and compromise posterior-circulation perfusion, its presence could be associated with adverse outcomes even when no perioperative neurologic event occurs [11–15]. The relative associations of severe carotid and vertebral stenosis with long-term mortality after isolated OPCABG remain incompletely characterized.

The present study therefore evaluated the association between severe extracranial cerebrovascular stenosis and long-term all-cause mortality after isolated OPCABG. We separately assessed severe carotid and vertebral artery disease, described their overlap, and examined early postoperative cerebral infarction and 30-day mortality.

## Methods

### Study design and population

**Fig. 1:**
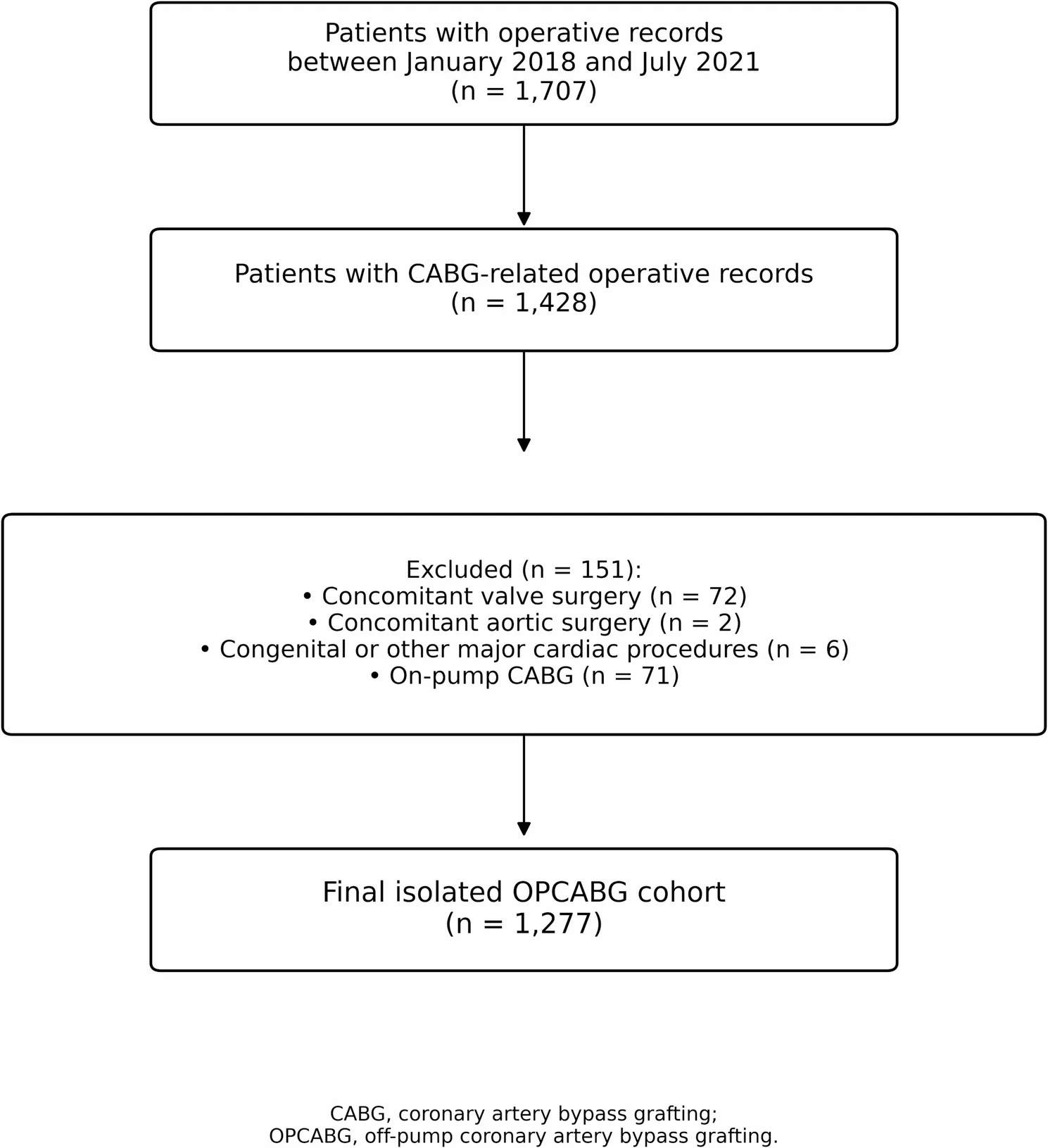
Study flow diagram. CABG, coronary artery bypass grafting; OPCABG, off-pump coronary artery bypass grafting

This retrospective cohort study included consecutive patients with CABG-related operative records at Beijing Anzhen Hospital between January 2018 and July 2021. During this period, 1,707 patients had operative records, of whom 1,428 had CABG-related procedures. We excluded 72 patients undergoing concomitant valve surgery, 2 undergoing concomitant aortic surgery, 6 undergoing congenital or other major cardiac procedures, and 71 undergoing on-pump CABG. The final cohort comprised 1,277 patients undergoing isolated OPCABG (Fig. 1). All included procedures were performed by a single experienced surgeon.

At our center, OPCABG was the preferred revascularization strategy when technically feasible. Conventional on-pump CABG was used when cardiopulmonary bypass was judged necessary because of operative complexity, hemodynamic considerations, or the need for concomitant cardiac procedures. Patients with prior CABG, concomitant major cardiac surgery, or incomplete key clinical or follow-up data were excluded.

The study was approved by the Institutional Review Board of Beijing Anzhen Hospital (approval no. 2026046X), which waived the requirement for individual informed consent because of the retrospective design. The study was conducted in accordance with the Declaration of Helsinki.

## Operative strategy

OPCABG was performed through a median sternotomy using mechanical cardiac stabilization and standard coronary anastomotic techniques. Conduit selection and graft configuration were determined by coronary anatomy and surgeon judgment. Aortic manipulation was minimized when feasible. When a proximal anastomosis of a venous graft to the ascending aorta was required, a clampless proximal anastomotic device (Heartstring) was used in most cases; exact device use was not captured as a structured variable. Operative duration was recorded in whole hours in the source database, and minute-level values were therefore unavailable.

No patient underwent carotid or vertebral artery revascularization before CABG or during the index hospitalization. Cerebrovascular interventions after discharge were not systematically captured. Nine patients (0.7%) had a documented history of paroxysmal atrial fibrillation; none underwent concomitant surgical ablation, pulmonary vein isolation, a Maze procedure, or left atrial appendage intervention.

## Assessment of cerebrovascular stenosis

All patients underwent preoperative duplex ultrasonography of the bilateral carotid and vertebral arteries. Stenosis severity was determined from the final vascular ultrasound report using an integrated assessment of B-mode morphology, color-flow imaging, and spectral Doppler findings. Stenosis was categorized as none or mild (< 50% diameter reduction), moderate (50%–69%), severe (70%–99%), or occlusion (100%).

For the primary analysis, severe cerebrovascular stenosis was defined as severe stenosis or occlusion involving at least one extracranial carotid or vertebral artery. For patients with multivessel involvement, the most severe lesion determined the overall category. Carotid and vertebral lesions were also analyzed separately. Reports describing possible occlusion without confirmatory evidence were reviewed individually and were not classified as definite occlusion unless supported by repeat ultrasonography or additional imaging.

## Clinical variables

Baseline variables included age, sex, hypertension, diabetes mellitus, prior myocardial infarction, prior cerebrovascular disease, hyperlipidemia, left ventricular ejection fraction (LVEF), and body mass index (BMI). Prior cerebrovascular disease was defined from the medical record as a documented history of cerebral infarction, intracranial hemorrhage, transient ischemic attack, or another clinically diagnosed cerebrovascular disorder. Operative variables included duration, number of distal anastomoses, coronary endarterectomy, and graft strategy.

## Follow-up and outcomes

Patients were followed from the date of OPCABG until death or administrative censoring on December 31, 2024. Survival status and causes of death were obtained from institutional records and linked follow-up databases. The primary outcome was long-term all-cause mortality. Causes of death were categorized as cardiac, cerebrovascular, or noncardiac and noncerebrovascular and were analyzed descriptively because of the limited number of cause-specific events.

Early outcomes included in-hospital postoperative cerebral infarction and 30-day all-cause mortality. Postoperative cerebral infarction was defined as a newly diagnosed cerebral infarction occurring after surgery during the index hospitalization. Thirty-day mortality was defined as death from any cause within 30 days after surgery. Long-term major adverse cardiac and cerebrovascular events were not systematically captured and were therefore not analyzed.

### Statistical analysis

Continuous variables are presented as median [interquartile range] and were compared using the Mann–Whitney U test. Categorical variables are presented as number (percentage) and were compared using the chi-square test or Fisher exact test, as appropriate. Kaplan–Meier methods were used to estimate survival, with comparisons by the log-rank test. Numbers at risk and censoring marks are shown on the revised survival curves.

Primary multivariable Cox proportional hazards models were adjusted a priori for age, sex, hypertension, diabetes mellitus, prior myocardial infarction, prior cerebrovascular disease, hyperlipidemia, LVEF, and BMI. Covariates were selected on the basis of clinical relevance and established CABG literature rather than univariable P values. Separate models evaluated severe carotid stenosis, severe vertebral stenosis, and any severe cerebrovascular stenosis; a mutual model included both carotid and vertebral variables. Proportional hazards assumptions were assessed using Schoenfeld residuals and were not violated.

Because only 87 deaths occurred, additional parsimonious and ridge-penalized Cox models were used as sensitivity analyses to assess potential overfitting. Exploratory subgroup analyses for any severe cerebrovascular stenosis were stratified by age (< 70 vs. ≥ 70 years), sex, and LVEF (< 50% vs. ≥ 50%) and used parsimonious Cox models adjusted for age, sex, diabetes mellitus, prior cerebrovascular disease, and LVEF, except that the subgroup-defining variable was omitted from the corresponding model. Interaction was assessed by including multiplicative exposure-by-subgroup terms. Analyses used complete cases for the variables included in each model. Two-sided *P* < 0.05 was considered statistically significant. Analyses were performed using Python version 3.11 (Python Software Foundation, Wilmington, DE, USA) with the pandas, SciPy, matplotlib, and lifelines packages.

## Results

### Study cohort and baseline characteristics

The study flow is shown in Fig. 1. Of 1,428 CABG-related operative records, 151 were excluded, leaving 1,277 patients undergoing isolated OPCABG. The median follow-up was 4.79 years, and 87 deaths occurred.

Severe cerebrovascular stenosis was present in 186 patients (14.6%). Severe carotid stenosis or occlusion was present in 100 patients (7.8%), and severe vertebral stenosis or occlusion in 109 (8.5%). Among the 186 patients with any severe disease, 77 had isolated severe carotid disease, 86 had isolated severe vertebral disease, and 23 had severe disease in both territories.

Baseline and operative characteristics are summarized in Table 1. Patients with severe cerebrovascular stenosis were older and more frequently had hypertension, diabetes mellitus, and prior cerebrovascular disease. Coronary endarterectomy was also more common in the severe-stenosis group. Other operative characteristics and graft strategies were broadly comparable.

**Table 1:** Baseline and operative characteristics according to severe cerebrovascular stenosis

Variable	No severe stenosis (*n* = 1,091)	Any severe stenosis (*n* = 186)	*P* value
Baseline characteristics			
Age, years	62.0 [55.0–67.0]	65.0 [61.0–69.0]	< 0.001
Male sex	848 (77.7%)	151 (81.2%)	0.337
Hypertension	687 (63.0%)	143 (76.9%)	< 0.001
Diabetes mellitus	401 (36.8%)	89 (47.8%)	0.005
Prior myocardial infarction	232 (21.3%)	34 (18.3%)	0.407
Prior cerebrovascular disease	74 (6.8%)	41 (22.0%)	< 0.001
Hyperlipidemia	529 (48.5%)	93 (50.0%)	0.763
LVEF, %	61.0 [57.0–65.0]	61.0 [55.0–65.0]	0.671
BMI, kg/m²	25.40 [23.56–27.61]	25.40 [23.39–27.34]	0.417
Operative characteristics			
Operative time, hours	4.0 [4.0–5.0]	4.0 [4.0–5.0]	0.322
Number of distal anastomoses	4.0 [4.0–4.0]	4.0 [4.0–4.0]	0.535
Coronary endarterectomy	57 (5.2%)	21 (11.3%)	0.002
Bypass graft type			0.135
Arterial grafts only	10 (0.9%)	2 (1.1%)	
Venous grafts only	315 (28.9%)	68 (36.6%)	
Combined arterial and venous grafts	748 (68.6%)	115 (61.8%)	
Bilateral internal mammary artery grafting	18 (1.6%)	1 (0.5%)	

Values are median [interquartile range] or n (%). BMI, body mass index; LVEF, left ventricular ejection fraction.

### Early postoperative outcomes

Early outcomes are summarized in Supplementary Table S1. In-hospital postoperative cerebral infarction occurred in 17 patients (1.33%) and was more frequent among patients with any severe cerebrovascular stenosis than among those without severe disease (11/186 [5.91%] vs. 6/1,091 [0.55%]; *P* < 0.001). Thirty-day all-cause mortality occurred in 27 patients (2.11%) and was also higher in the severe-stenosis group (9/186 [4.84%] vs. 18/1,091 [1.65%]; *P* = 0.011). Among the 27 deaths within 30 days, 20 (74.1%) were cardiac, 2 (7.4%) cerebrovascular, and 5 (18.5%) noncardiac and noncerebrovascular.

### Kaplan–Meier survival analysis

**Fig. 2:**
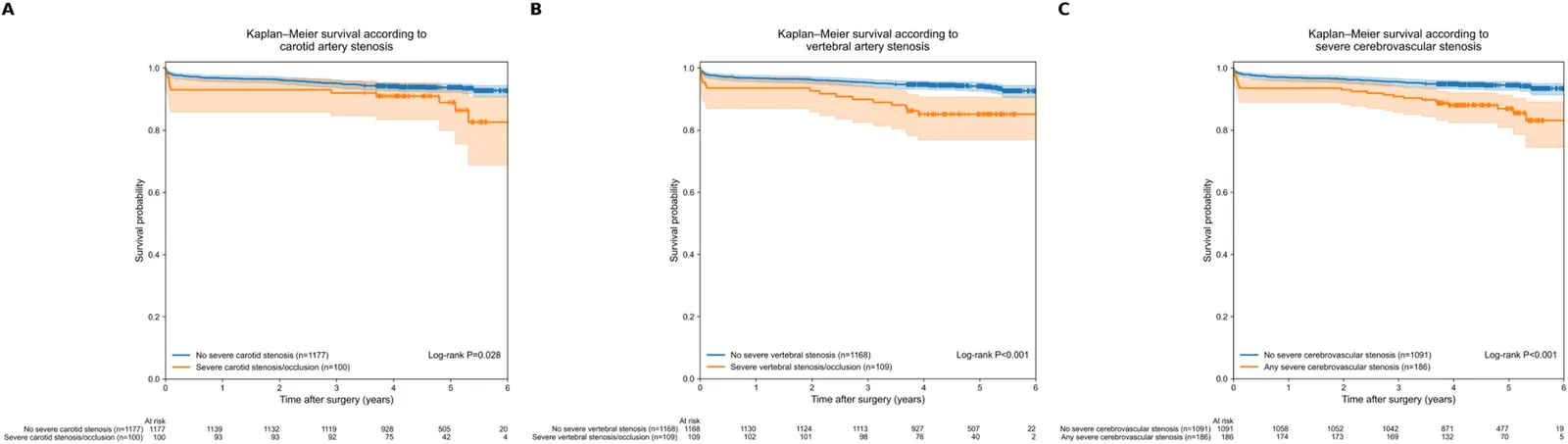
Kaplan–Meier survival curves according to cerebrovascular stenosis. (**A**) Severe carotid artery stenosis or occlusion versus no severe carotid disease. (**B**) Severe vertebral artery stenosis or occlusion versus no severe vertebral disease. (C) Any severe cerebrovascular stenosis versus no severe disease. Shaded areas indicate 95% confidence intervals. Censoring marks and numbers at risk are shown. P values were calculated using the log-rank test

Kaplan–Meier curves are presented in Fig. 2. Severe carotid stenosis or occlusion was associated with lower unadjusted survival (log-rank *P* = 0.028). Survival was also significantly lower among patients with severe vertebral stenosis or occlusion (*P* < 0.001) and among those with any severe cerebrovascular stenosis (*P* < 0.001).

### Multivariable cox regression

**Fig. 3:**
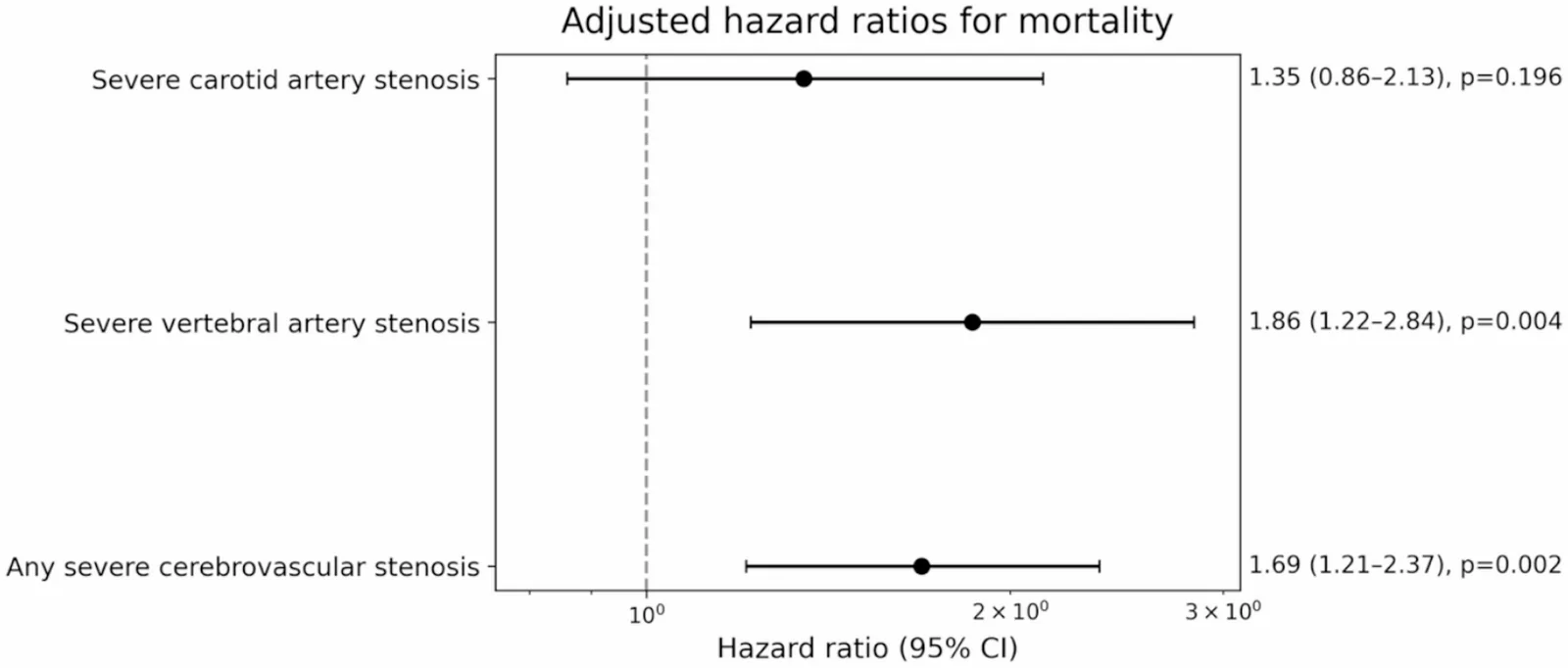
Adjusted hazard ratios for long-term all-cause mortality according to severe cerebrovascular stenosis. Hazard ratios and 95% confidence intervals were derived from multivariable Cox models adjusted for age, sex, hypertension, diabetes mellitus, prior myocardial infarction, prior cerebrovascular disease, hyperlipidemia, LVEF, and BMI

Multivariable Cox results are shown in Table 2; Fig. 3. Severe carotid stenosis was not independently associated with mortality (HR, 1.35; 95% CI, 0.86–2.13; *P* = 0.196). In contrast, severe vertebral stenosis remained associated with mortality (HR, 1.86; 95% CI, 1.22–2.84; *P* = 0.004), as did any severe cerebrovascular stenosis (HR, 1.69; 95% CI, 1.21–2.37; *P* = 0.002). In the mutual model, vertebral stenosis remained associated with mortality (HR, 1.82; 95% CI, 1.18–2.78; *P* = 0.006), whereas carotid stenosis did not (HR, 1.26; 95% CI, 0.80–2.00; *P* = 0.319). Results were directionally consistent in parsimonious and ridge-penalized sensitivity analyses (Supplementary Table S3), and no evidence of violation of the proportional hazards assumption was detected in the fully adjusted models (Supplementary Table S4). Full multivariable Cox regression outputs, including all covariates, are provided in Supplementary Table S5. Additional adjustment for coronary endarterectomy did not materially alter the associations of any severe cerebrovascular stenosis, severe carotid artery stenosis, or severe vertebral artery stenosis with long-term mortality.

**Table 2:** Multivariable Cox regression analyses for long-term all-cause mortality

Model	Exposure	HR	95% CI	*P* value
Model A	Severe carotid artery stenosis	1.35	0.86–2.13	0.196
Model B	Severe vertebral artery stenosis	1.86	1.22–2.84	0.004
Model C	Any severe cerebrovascular stenosis	1.69	1.21–2.37	0.002
Mutual model	Severe carotid artery stenosis	1.26	0.80–2.00	0.319
	Severe vertebral artery stenosis	1.82	1.18–2.78	0.006

Models were adjusted for age, sex, hypertension, diabetes mellitus, prior myocardial infarction, prior cerebrovascular disease, hyperlipidemia, LVEF, and BMI. The mutual model included both carotid and vertebral stenosis.

### Subgroup analysis

**Fig. 4:**
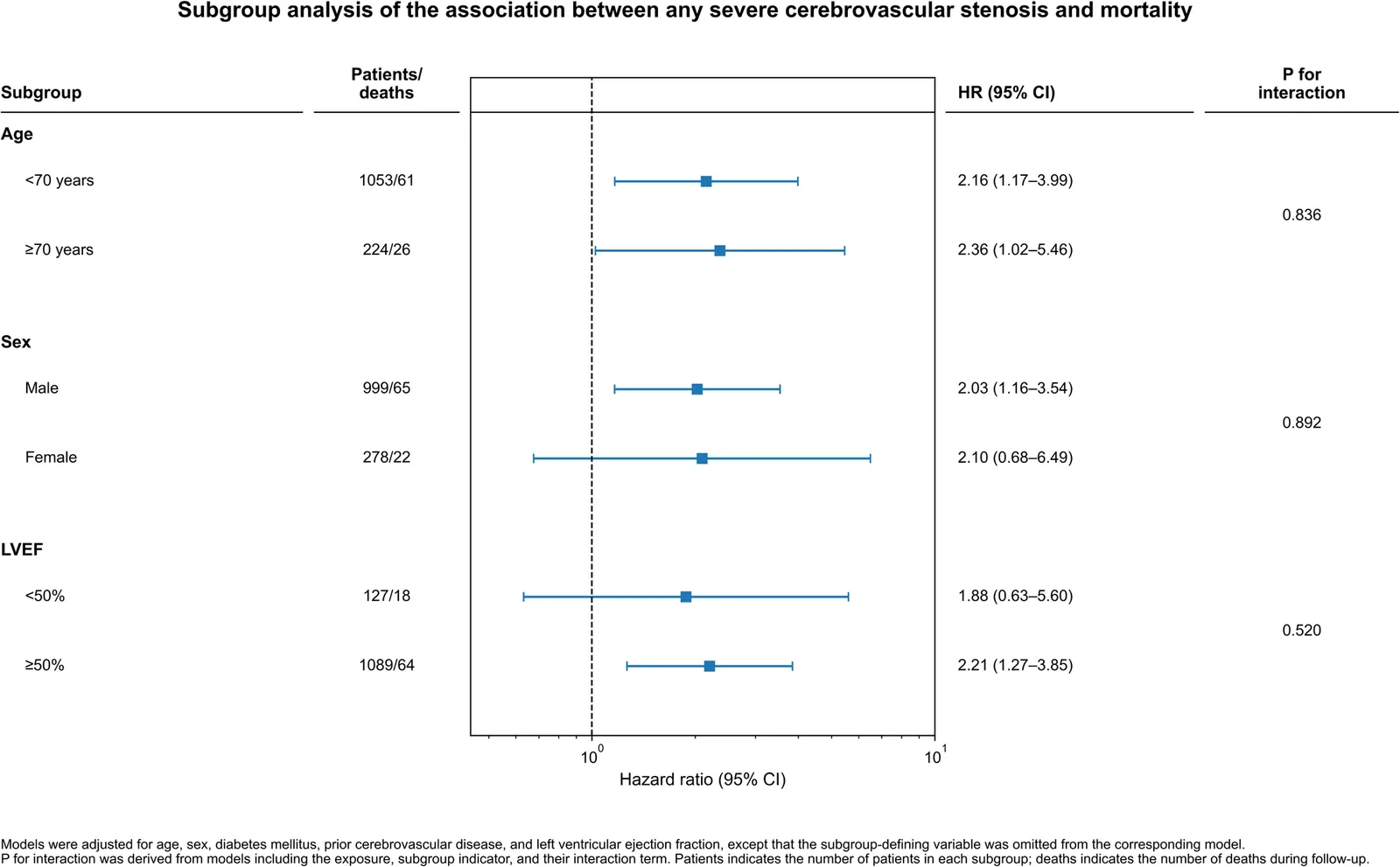
Exploratory subgroup analysis of the association between any severe cerebrovascular stenosis and long-term all-cause mortality. Parsimonious Cox models were adjusted for age, sex, diabetes mellitus, prior cerebrovascular disease, and LVEF, except that the subgroup-defining variable was omitted from the corresponding model. Patients/deaths indicates the number of patients and deaths in each subgroup. P values for interaction were derived from models including the exposure, subgroup indicator, and their multiplicative interaction term

Exploratory subgroup analyses are shown in Fig. 4. The association between any severe cerebrovascular stenosis and mortality was similar in patients aged < 70 years (HR, 2.16; 95% CI, 1.17–3.99) and ≥ 70 years (HR, 2.36; 95% CI, 1.02–5.46; P for interaction = 0.836). Findings were also directionally consistent by sex (P for interaction = 0.892) and LVEF category (P for interaction = 0.520). No statistically significant interaction was observed.

### Exploratory severity-stratified analysis

To further explore a potential dose-response relationship, cerebrovascular stenosis was additionally categorized into three severity levels: none or mild, moderate, and severe or occlusion. Survival differed significantly across the three categories (log-rank *P* < 0.001). Compared with patients with none or mild stenosis, moderate stenosis was not associated with mortality after parsimonious adjustment (HR, 1.01; 95% CI, 0.56–1.82; *P* = 0.985), whereas severe stenosis or occlusion was associated with higher mortality (HR, 2.10; 95% CI, 1.25–3.54; *P* = 0.005). The adjusted trend across increasing severity categories was statistically significant (HR per one-category increase, 1.41; 95% CI, 1.08–1.85; P for trend = 0.011; Supplementary Table S6 and Supplementary Figure S1).

### Causes of death

Causes of death are summarized in Supplementary Table S2. Of the 87 deaths during follow-up, 51 (58.6%) were cardiac, 11 (12.6%) cerebrovascular, and 25 (28.7%) noncardiac and noncerebrovascular. Cause-specific outcomes were not modeled because of the limited number of events.

## Discussion

In this single-center cohort of patients undergoing isolated OPCABG, severe extracranial cerebrovascular stenosis was associated with both adverse early postoperative outcomes and increased long-term mortality. The principal findings were that (1) patients with severe disease had higher rates of in-hospital postoperative cerebral infarction and 30-day mortality; (2) severe vertebral stenosis and any severe cerebrovascular stenosis were independently associated with long-term all-cause mortality; and (3) the association of any severe cerebrovascular stenosis with mortality was directionally consistent across age, sex, and LVEF subgroups.

Preoperative cerebrovascular assessment in CABG has traditionally emphasized carotid disease because of its established relationship with perioperative stroke [1–9]. Recent studies have continued to refine risk stratification and long-term outcome assessment after coronary artery bypass grafting, highlighting the importance of individualized prognostic evaluation in contemporary CABG practice. [16–17] The present study extends this literature by evaluating both carotid and vertebral territories within a uniform isolated OPCABG cohort and by focusing on long-term mortality rather than perioperative stroke alone. Our findings confirm that severe carotid disease identifies patients with worse unadjusted survival and a substantially higher incidence of in-hospital cerebral infarction. However, the long-term mortality association was attenuated after adjustment, suggesting that carotid disease may partly reflect age and vascular comorbidity rather than exerting an independent effect on late mortality. The exploratory severity-stratified analysis further supported a graded association between cerebrovascular disease burden and long-term mortality. However, this pattern was not strictly linear, because patients with moderate stenosis had a risk like those with none or mild stenosis, whereas the excess risk was primarily observed in patients with severe stenosis or occlusion. These findings suggest that only advanced cerebrovascular disease, rather than moderate stenosis, identifies patients at particularly high long-term risk.

The stronger association observed for vertebral artery disease is clinically noteworthy but should be interpreted cautiously. One possible explanation is that vertebral stenosis marks a more diffuse and advanced systemic atherosclerotic phenotype [11, 12]. Posterior-circulation disease could also be associated with impaired cerebrovascular reserve or increased susceptibility to hemodynamic and embolic injury [13–15]. These mechanisms were not directly evaluated in this study and remain speculative. We did not measure cerebral perfusion, collateral status, embolic burden, or cerebrovascular reserve, and the observed association does not establish a causal pathway.

The early outcome findings provide additional clinical context. Patients with severe cerebrovascular stenosis had markedly higher rates of postoperative cerebral infarction and 30-day death. Nevertheless, the study was designed primarily to evaluate long-term all-cause mortality, and long-term MACCE events were not systematically captured. The early results should therefore be regarded as descriptive and hypothesis-generating rather than as evidence that preoperative cerebrovascular imaging should directly trigger a specific intervention.

The absence of significant interaction across age, sex, and LVEF suggests that the association was not confined to a single readily identifiable subgroup. However, subgroup event counts were limited, confidence intervals were wide in women and patients with reduced LVEF, and these analyses were exploratory. The parsimonious adjustment strategy was chosen to reduce model instability, but the subgroup estimates should not be interpreted as definitive evidence of homogeneity.

The clinical implications are primarily related to risk characterization. Routine bilateral carotid and vertebral duplex imaging may identify patients with a greater systemic atherosclerotic burden who warrant careful perioperative neurologic assessment and intensified long-term secondary prevention. However, we did not perform formal incremental prediction analyses using C-index improvement, time-dependent area under the curve, net reclassification improvement, or integrated discrimination improvement. Accordingly, the present findings demonstrate association rather than added predictive value.

Several limitations should be acknowledged. First, this was a retrospective single-center study involving procedures performed by one surgeon, which may limit generalizability and does not eliminate residual confounding. Second, stenosis severity was based on final clinical ultrasound reports rather than centralized blinded re-reading, and intracranial arterial stenosis, distal vertebrobasilar disease, collateral circulation, and cerebral perfusion were not systematically evaluated. Third, the limited number of deaths constrained model complexity and subgroup precision, although parsimonious and penalized sensitivity analyses supported the principal direction of association. Fourth, complete EuroSCORE II and Society of Thoracic Surgeons risk-score variables were unavailable, precluding reliable score reconstruction. Long-term medication use and adherence were not systematically recorded. Completeness of revascularization was not captured as a structured variable. Coronary endarterectomy was available and was included in an additional sensitivity analysis; further adjustment for endarterectomy did not materially alter the principal associations. Fifth, the exact use of Heartstring and other aortic no-touch strategies was not prospectively coded. Sixth, post-discharge carotid or vertebral interventions and long-term nonfatal MACCE events were not systematically captured. Finally, the observational design precludes causal inference.

## Conclusion

Severe extracranial cerebrovascular stenosis was associated with higher early neurologic event rates and increased long-term all-cause mortality after isolated OPCABG. Severe vertebral artery stenosis showed the strongest association and may serve as a marker of advanced systemic atherosclerotic disease. Prospective multicenter studies incorporating standardized vascular imaging, cerebral hemodynamic assessment, and comprehensive clinical follow-up are needed to validate these findings and determine their clinical utility.

## Supplementary Information

Supplementary Material 1

Supplementary Material 2

Supplementary Material 3

## Data Availability

No datasets were generated or analysed during the current study.

## Declarations

### **Generative AI and AI-Assisted Technologie**

During preparation of the manuscript, the authors used ChatGPT (OpenAI) for language editing and manuscript organization. The authors reviewed and revised all content and take full responsibility for the final manuscript.
