# Supplementary material for: Cerebrovascular stenosis and mortality after isolated off-pump coronary artery bypass grafting
Source: J Cardiothorac Surg. 2026 Jul 29;21:856. doi: 10.1186/s13019-026-04664-y (PMC13717997; doi:10.1186/s13019-026-04664-y)
Supplement: Supplementary file 1 [file 13019_2026_4664_MOESM1_ESM.docx]

**Supplementary Table S1. Proportional hazards assumption testing for the Cox model of any severe cerebrovascular stenosis.**

| Covariate | Test Statistic | P Value |
| --- | --- | --- |
| Any severe | 0.257 | 0.612 |
| Age, years | 0.316 | 0.574 |
| Sex male | 1.938 | 0.164 |
| BMI | 0.023 | 0.879 |
| LVEF, % | 0.023 | 0.880 |
| Prior myocardial infarction | 2.719 | 0.099 |
| Prior cerebrovascular disease | 0.004 | 0.952 |
| Diabetes mellitus | 0.003 | 0.954 |
| Hyperlipidemia | 0.626 | 0.429 |
| Hypertension | 0.017 | 0.895 |

Proportional hazards assumptions were evaluated using Schoenfeld residual tests. A P value > 0.05 was considered consistent with no evidence of violation of the proportional hazards assumption.
