# Supplementary material for: Cerebrovascular stenosis and mortality after isolated off-pump coronary artery bypass grafting
Source: J Cardiothorac Surg. 2026 Jul 29;21:856. doi: 10.1186/s13019-026-04664-y (PMC13717997; doi:10.1186/s13019-026-04664-y)
Supplement: Supplementary file 2 [file 13019_2026_4664_MOESM2_ESM.docx]

**Supplementary Table S2. Full multivariable Cox regression results for the carotid, vertebral, any severe, and mutual models.**

| **Model A:Severe carotid artery stenosis** | | | | |
| --- | --- | --- | --- | --- |
| Covariate | HR | 95% CI Lower | **95% CI Upper** | **P Value** |
| Severe carotid artery stenosis | 1.351 | 0.856 | 2.133 | 0.196 |
| Age, years | 1.025 | 1.01 | 1.04 | 0.001 |
| sex_male | 0.912 | 0.675 | 1.232 | 0.548 |
| BMI, kg/m² | 0.996 | 0.958 | 1.034 | 0.825 |
| LVEF, % | 0.969 | 0.954 | 0.985 | <0.001 |
| Prior myocardial infarction | 1.018 | 0.758 | 1.366 | 0.905 |
| Prior cerebrovascular disease | 1.415 | 0.916 | 2.186 | 0.118 |
| Diabetes mellitus | 1.188 | 0.918 | 1.537 | 0.189 |
| Hyperlipidemia | 1.115 | 0.872 | 1.426 | 0.384 |
| Hypertension | 1.053 | 0.817 | 1.356 | 0.69 |
| **Model B: Severe vertebral artery stenosis** | | | | |
| Covariate | HR | 95% CI Lower | **95% CI Upper** | **P Value** |
| Severe vertebral artery stenosis | 1.863 | 1.224 | 2.837 | 0.004 |
| Age, years | 1.025 | 1.01 | 1.041 | <0.001 |
| sex_male | 0.908 | 0.672 | 1.226 | 0.528 |
| BMI, kg/m² | 0.995 | 0.957 | 1.034 | 0.782 |
| LVEF, % | 0.968 | 0.953 | 0.984 | <0.001 |
| Prior myocardial infarction | 1.02 | 0.761 | 1.367 | 0.894 |
| Prior cerebrovascular disease | 1.381 | 0.915 | 2.085 | 0.125 |
| Diabetes mellitus | 1.189 | 0.92 | 1.536 | 0.185 |
| Hyperlipidemia | 1.117 | 0.873 | 1.428 | 0.38 |
| Hypertension | 1.039 | 0.807 | 1.337 | 0.766 |
| **Model C: Any severe cerebrovascular stenosis** | | | | |
| Covariate | HR | 95% CI Lower | **95% CI Upper** | **P Value** |
| Any severe cerebrovascular stenosis | 1.695 | 1.21 | 2.374 | 0.002 |
| Age, years | 1.024 | 1.009 | 1.04 | 0.002 |
| sex_male | 0.902 | 0.667 | 1.219 | 0.501 |
| BMI, kg/m² | 0.995 | 0.958 | 1.034 | 0.814 |
| LVEF, % | 0.969 | 0.953 | 0.985 | <0.001 |
| Prior myocardial infarction | 1.025 | 0.764 | 1.375 | 0.871 |
| Prior cerebrovascular disease | 1.349 | 0.889 | 2.048 | 0.159 |
| Diabetes mellitus | 1.179 | 0.912 | 1.523 | 0.208 |
| Hyperlipidemia | 1.115 | 0.872 | 1.426 | 0.384 |
| Hypertension | 1.038 | 0.806 | 1.335 | 0.774 |
| Mutual model: Severe carotid and severe vertebral artery stenosis | | | | |
| Covariate | HR | 95% CI Lower | **95% CI Upper** | **P Value** |
| Severe carotid artery stenosis | 1.263 | 0.798 | 2 | 0.319 |
| Severe vertebral artery stenosis | 1.816 | 1.185 | 2.784 | 0.006 |
| Age, years | 1.025 | 1.01 | 1.04 | 0.001 |
| sex_male | 0.906 | 0.67 | 1.224 | 0.519 |
| BMI, kg/m² | 0.996 | 0.958 | 1.034 | 0.818 |
| LVEF, % | 0.968 | 0.953 | 0.984 | <0.001 |
| Prior myocardial infarction | 1.024 | 0.763 | 1.375 | 0.874 |
| Prior cerebrovascular disease | 1.354 | 0.894 | 2.051 | 0.153 |
| Diabetes mellitus | 1.184 | 0.916 | 1.53 | 0.196 |
| Hyperlipidemia | 1.114 | 0.871 | 1.425 | 0.389 |
| Hypertension | 1.036 | 0.805 | 1.332 | 0.786 |

All models were adjusted for age, sex, hypertension, diabetes mellitus, prior myocardial infarction, prior cerebrovascular disease, hyperlipidemia, left ventricular ejection fraction, and body mass index. The mutual model included both severe carotid artery stenosis and severe vertebral artery stenosis simultaneously.
