# Supplementary material for: Cerebrovascular stenosis and mortality after isolated off-pump coronary artery bypass grafting
Source: J Cardiothorac Surg. 2026 Jul 29;21:856. doi: 10.1186/s13019-026-04664-y (PMC13717997; doi:10.1186/s13019-026-04664-y)
Supplement: Supplementary file 3 [file 13019_2026_4664_MOESM3_ESM.docx]

**Supplementary Material**

**Supplementary Table S1. Early postoperative outcomes according to severe cerebrovascular stenosis**

| Outcome | Overall (n=1,277) | No severe stenosis (n=1,091) | Any severe stenosis (n=186) | P value |
| --- | --- | --- | --- | --- |
| In-hospital postoperative cerebral infarction | 17 (1.33%) | 6 (0.55%) | 11 (5.91%) | <0.001 |
| 30-day all-cause mortality | 27 (2.11%) | 18 (1.65%) | 9 (4.84%) | 0.011 |

*Values are n (%). P values were calculated using Fisher exact tests. Postoperative cerebral infarction was defined as a newly diagnosed cerebral infarction during the index hospitalization after surgery. Thirty-day mortality was defined as death from any cause within 30 days after surgery.*

**Supplementary Table S2. Causes of death during follow-up**

| Cause-of-death category | No severe stenosis | Any severe stenosis | Overall, n (%) |
| --- | --- | --- | --- |
| Cardiac | 36 | 15 | 51 (58.6%) |
| Cerebrovascular | 5 | 6 | 11 (12.6%) |
| Noncardiac and noncerebrovascular | 21 | 4 | 25 (28.7%) |
| Total | 62 | 25 | 87 (100%) |

*Percentages were calculated among all deaths. Causes of death were summarized descriptively because of the limited number of cause-specific events.*

**Supplementary Table S3. Sensitivity analyses of multivariable Cox models**

| **Exposure** | **Parsimonious Cox model** | | **Ridge-penalized Cox model*** | |
| --- | --- | --- | --- | --- |
|  | **HR (95% CI)** | **P value** | **HR (95% CI)** | **P value** |
| Any severe cerebrovascular stenosis | 2.10 (1.282–3.441) | 0.003 | 1.70 (1.110–2.595) | 0.015 |
| Severe carotid artery stenosis/occlusion | 1.50 (0.794–2.823) | 0.212 | 1.49 (0.863–2.571) | 0.153 |
| Severe vertebral artery stenosis/occlusion | 2.45 (1.383–4.345) | 0.002 | 1.77 (1.052–2.990) | 0.031 |
| Severe carotid artery stenosis/occlusion (mutual model) | 1.34 (0.706–2.541) | 0.371 | 1.41 (0.819–2.441) | 0.214 |
| Severe vertebral artery stenosis/occlusion (mutual model) | 2.37 (1.326–4.222) | 0.004 | 1.72 (1.018–2.900) | 0.043 |

*The ridge-penalized Cox model used an L2 penalizer of 0.05 and the fully adjusted covariate set. The parsimonious model was adjusted for age, sex, diabetes mellitus, prior cerebrovascular disease, and left ventricular ejection fraction.

**Supplementary Table S4. Proportional hazards assumption testing for the fully adjusted Cox models.**

| **Model** | **Covariate** | **Test statistic** | **P value** | **Assessment** |
| --- | --- | --- | --- | --- |
| Model A: Any severe cerebrovascular stenosis | Body mass index | 0.121 | 0.728 | No evidence of violation |
|  | Left ventricular ejection fraction | 0.069 | 0.793 | No evidence of violation |
|  | Any severe cerebrovascular stenosis | 0.434 | 0.510 | No evidence of violation |
|  | Age, years | 0.222 | 0.638 | No evidence of violation |
|  | Prior myocardial infarction | 2.050 | 0.152 | No evidence of violation |
|  | Prior cerebrovascular disease | 0.007 | 0.934 | No evidence of violation |
|  | Male sex | 1.244 | 0.265 | No evidence of violation |
|  | Diabetes mellitus | 0.011 | 0.916 | No evidence of violation |
|  | Hyperlipidemia | 0.833 | 0.361 | No evidence of violation |
|  | Hypertension | 0.046 | 0.830 | No evidence of violation |
| Model B: Severe carotid artery stenosis/occlusion | Body mass index | 0.132 | 0.716 | No evidence of violation |
|  | Left ventricular ejection fraction | 0.083 | 0.773 | No evidence of violation |
|  | Age, years | 0.147 | 0.701 | No evidence of violation |
|  | Prior myocardial infarction | 2.127 | 0.145 | No evidence of violation |
|  | Prior cerebrovascular disease | 0.000 | 0.986 | No evidence of violation |
|  | Male sex | 1.408 | 0.235 | No evidence of violation |
|  | Diabetes mellitus | 0.006 | 0.938 | No evidence of violation |
|  | Severe carotid artery stenosis/occlusion | 0.009 | 0.926 | No evidence of violation |
|  | Hyperlipidemia | 0.888 | 0.346 | No evidence of violation |
|  | Hypertension | 0.066 | 0.797 | No evidence of violation |
| Model C: Severe vertebral artery stenosis/occlusion | Body mass index | 0.176 | 0.675 | No evidence of violation |
|  | Left ventricular ejection fraction | 0.126 | 0.723 | No evidence of violation |
|  | Age, years | 0.159 | 0.690 | No evidence of violation |
|  | Prior myocardial infarction | 2.214 | 0.137 | No evidence of violation |
|  | Prior cerebrovascular disease | 0.002 | 0.967 | No evidence of violation |
|  | Severe vertebral artery stenosis/occlusion | 0.276 | 0.599 | No evidence of violation |
|  | Male sex | 1.367 | 0.242 | No evidence of violation |
|  | Diabetes mellitus | 0.009 | 0.925 | No evidence of violation |
|  | Hyperlipidemia | 0.850 | 0.357 | No evidence of violation |
|  | Hypertension | 0.058 | 0.810 | No evidence of violation |
| Model D: Mutual carotid–vertebral model | Body mass index | 0.160 | 0.689 | No evidence of violation |
|  | Left ventricular ejection fraction | 0.135 | 0.714 | No evidence of violation |
|  | Age, years | 0.142 | 0.707 | No evidence of violation |
|  | Prior myocardial infarction | 2.223 | 0.136 | No evidence of violation |
|  | Prior cerebrovascular disease | 0.000 | 0.997 | No evidence of violation |
|  | Severe vertebral artery stenosis/occlusion | 0.273 | 0.601 | No evidence of violation |
|  | Male sex | 1.387 | 0.239 | No evidence of violation |
|  | Diabetes mellitus | 0.006 | 0.940 | No evidence of violation |
|  | Severe carotid artery stenosis/occlusion | 0.055 | 0.815 | No evidence of violation |
|  | Hyperlipidemia | 0.878 | 0.349 | No evidence of violation |
|  | Hypertension | 0.058 | 0.809 | No evidence of violation |

Proportional hazards assumptions were evaluated using Schoenfeld residual tests. A P value >0.05 was considered consistent with no evidence of violation of the proportional hazards assumption. Fully adjusted models included age, sex, hypertension, diabetes mellitus, prior myocardial infarction, prior cerebrovascular disease, hyperlipidemia, left ventricular ejection fraction, and body mass index. The mutual model included severe carotid and severe vertebral artery stenosis simultaneously.

**Supplementary Table S5. Full multivariable Cox regression results for the carotid, vertebral, any severe, and mutual models.**

| **Model A:Severe carotid artery stenosis** | | | | |
| --- | --- | --- | --- | --- |
| Covariate | HR | 95% CI Lower | **95% CI Upper** | **P Value** |
| Severe carotid artery stenosis | 1.351 | 0.856 | 2.133 | 0.196 |
| Age, years | 1.025 | 1.01 | 1.04 | 0.001 |
| sex_male | 0.912 | 0.675 | 1.232 | 0.548 |
| BMI, kg/m² | 0.996 | 0.958 | 1.034 | 0.825 |
| LVEF, % | 0.969 | 0.954 | 0.985 | <0.001 |
| Prior myocardial infarction | 1.018 | 0.758 | 1.366 | 0.905 |
| Prior cerebrovascular disease | 1.415 | 0.916 | 2.186 | 0.118 |
| Diabetes mellitus | 1.188 | 0.918 | 1.537 | 0.189 |
| Hyperlipidemia | 1.115 | 0.872 | 1.426 | 0.384 |
| Hypertension | 1.053 | 0.817 | 1.356 | 0.69 |
| **Model B: Severe vertebral artery stenosis** | | | | |
| Covariate | HR | 95% CI Lower | **95% CI Upper** | **P Value** |
| Severe vertebral artery stenosis | 1.863 | 1.224 | 2.837 | 0.004 |
| Age, years | 1.025 | 1.01 | 1.041 | <0.001 |
| sex_male | 0.908 | 0.672 | 1.226 | 0.528 |
| BMI, kg/m² | 0.995 | 0.957 | 1.034 | 0.782 |
| LVEF, % | 0.968 | 0.953 | 0.984 | <0.001 |
| Prior myocardial infarction | 1.02 | 0.761 | 1.367 | 0.894 |
| Prior cerebrovascular disease | 1.381 | 0.915 | 2.085 | 0.125 |
| Diabetes mellitus | 1.189 | 0.92 | 1.536 | 0.185 |
| Hyperlipidemia | 1.117 | 0.873 | 1.428 | 0.38 |
| Hypertension | 1.039 | 0.807 | 1.337 | 0.766 |
| **Model C: Any severe cerebrovascular stenosis** | | | | |
| Covariate | HR | 95% CI Lower | **95% CI Upper** | **P Value** |
| Any severe cerebrovascular stenosis | 1.695 | 1.21 | 2.374 | 0.002 |
| Age, years | 1.024 | 1.009 | 1.04 | 0.002 |
| sex_male | 0.902 | 0.667 | 1.219 | 0.501 |
| BMI, kg/m² | 0.995 | 0.958 | 1.034 | 0.814 |
| LVEF, % | 0.969 | 0.953 | 0.985 | <0.001 |
| Prior myocardial infarction | 1.025 | 0.764 | 1.375 | 0.871 |
| Prior cerebrovascular disease | 1.349 | 0.889 | 2.048 | 0.159 |
| Diabetes mellitus | 1.179 | 0.912 | 1.523 | 0.208 |
| Hyperlipidemia | 1.115 | 0.872 | 1.426 | 0.384 |
| Hypertension | 1.038 | 0.806 | 1.335 | 0.774 |
| Mutual model: Severe carotid and severe vertebral artery stenosis | | | | |
| Covariate | HR | 95% CI Lower | **95% CI Upper** | **P Value** |
| Severe carotid artery stenosis | 1.263 | 0.798 | 2 | 0.319 |
| Severe vertebral artery stenosis | 1.816 | 1.185 | 2.784 | 0.006 |
| Age, years | 1.025 | 1.01 | 1.04 | 0.001 |
| sex_male | 0.906 | 0.67 | 1.224 | 0.519 |
| BMI, kg/m² | 0.996 | 0.958 | 1.034 | 0.818 |
| LVEF, % | 0.968 | 0.953 | 0.984 | <0.001 |
| Prior myocardial infarction | 1.024 | 0.763 | 1.375 | 0.874 |
| Prior cerebrovascular disease | 1.354 | 0.894 | 2.051 | 0.153 |
| Diabetes mellitus | 1.184 | 0.916 | 1.53 | 0.196 |
| Hyperlipidemia | 1.114 | 0.871 | 1.425 | 0.389 |
| Hypertension | 1.036 | 0.805 | 1.332 | 0.786 |

All models were adjusted for age, sex, hypertension, diabetes mellitus, prior myocardial infarction, prior cerebrovascular disease, hyperlipidemia, left ventricular ejection fraction, and body mass index. The mutual model included severe carotid artery stenosis and severe vertebral artery stenosis simultaneously. HR indicates hazard ratio; CI, confidence interval; LVEF, left ventricular ejection fraction.

**Supplementary Table S6. Exploratory severity-stratified analysis of cerebrovascular stenosis and all-cause mortality**

| **Severity category** | **Patients** | **Deaths** | **Crude mortality, %** | **Unadjusted HR (95% CI)** | **P value** | **Parsimonious adjusted HR (95% CI)** | **P value** |
| --- | --- | --- | --- | --- | --- | --- | --- |
| None or mild | 842 | 45 | 5.34 | Reference | - | Reference | - |
| Moderate | 249 | 17 | 6.83 | 1.26 (0.72-2.20) | 0.418 | 1.01 (0.56-1.82) | 0.985 |
| Severe or occlusion | 186 | 25 | 13.44 | 2.65 (1.62-4.32) | <0.001 | 2.10 (1.25-3.54) | 0.005 |
| *P for linear trend* |  |  |  | 1.60 (1.25-2.05) | <0.001 | 1.41 (1.08-1.85) | 0.011 |

**Note.** Cerebrovascular stenosis severity was defined by the most severe lesion identified in either the carotid or vertebral arterial territory. The parsimonious model was adjusted for age, sex, diabetes mellitus, prior cerebrovascular disease, and left ventricular ejection fraction. The unadjusted analyses included 1,277 patients and 87 deaths; the adjusted complete-case analyses included 1,216 patients and 82 deaths. HR indicates hazard ratio; CI, confidence interval.

*Overall three-group log-rank test: P<0.001.*

**Supplementary Figure S1. Kaplan-Meier survival according to cerebrovascular stenosis severity**

**Figure legend.** Kaplan-Meier survival curves according to three severity categories of cerebrovascular stenosis: none or mild, moderate, and severe or occlusion. Tick marks indicate censored observations, and numbers at risk are shown below the plot. Survival differed across severity categories (log-rank P<0.001).
